# Full daily re-optimization improves plan quality during online adaptive radiotherapy

**DOI:** 10.1016/j.phro.2024.100534

**Published:** 2024-01-10

**Authors:** Benjamin Tengler, Luise A. Künzel, Markus Hagmüller, David Mönnich, Simon Boeke, Daniel Wegener, Cihan Gani, Daniel Zips, Daniela Thorwarth

**Affiliations:** a Section for Biomedical Physics. Department of Radiation Oncology, University Hospital and Medical Faculty, Eberhard Karls University Tübingen, Germany; b National Center for Tumor Diseases (NCT), Dresden, Germany: German Cancer Research Center (DKFZ), Heidelberg, Germany, Faculty of Medicine and University Hospital Carl Gustav Carus, Technische Universität Dresden, Dresden, Germany, Helmholtz-Zentrum Dresden - Rossendorf (HZDR), Dresden, Germany; c Department of Radiotherapy and Radiation Oncology, Faculty of Medicine and University Hospital Carl Gustav Carus, Technische Universität Dresden, Dresden, Germany; d OncoRay – National Center for Radiation Research in Oncology, Faculty of Medicine and University Hospital Carl Gustav Carus, Technische Universität Dresden, Helmholtz-Zentrum Dresden - Rossendorf, Dresden, Germany; e Department of Radiation Oncology, University Hospital and Medical Faculty, Eberhard Karls University Tübingen, Germany

**Keywords:** Adaptive treatment planning, MRI guided radiotherapy, Online plan optimization, MR-Linac

## Abstract

**Background and purpose:**

Daily online treatment plan adaptation requires a fast workflow and planning process. Current online planning consists of adaptation of a predefined reference plan, which might be suboptimal in cases of large anatomic changes. The aim of this study was to investigate plan quality differences between the current online re-planning approach and a complete re-optimization.

**Material and methods:**

Magnetic resonance linear accelerator reference plans for ten prostate cancer patients were automatically generated using particle swarm optimization (PSO). Adapted plans were created for each fraction using (1) the current re-planning approach and (2) full PSO re-optimization and evaluated overall compliance with institutional dose-volume criteria compared to (3) clinically delivered fractions. Relative volume differences between reference and daily anatomy were assessed for planning target volumes (PTV60, PTV57.6), rectum and bladder and correlated with dose-volume results.

**Results:**

The PSO approach showed significantly higher adherence to dose-volume criteria than the reference approach and clinical fractions (p < 0.001). In 74 % of PSO plans at most one criterion failed compared to 56 % in the reference approach and 41 % in clinical plans. A fair correlation between PTV60 D98% and relative bladder volume change was observed for the reference approach. Bladder volume reductions larger than 50 % compared to the reference plan recurrently decreased PTV60 D98% below 56 Gy.

**Conclusion:**

Complete re-optimization maintained target coverage and organs at risk sparing even after large anatomic variations. Re-planning based on daily magnetic resonance imaging was sufficient for small variations, while large variations led to decreasing target coverage and organ-at-risk sparing.

## 1 Introduction

The goal of image-guided radiotherapy (IGRT) is to increase application accuracy by utilizing daily imaging prior to each treatment fraction [1]. Typically, IGRT is used to control and adjust translational shifts in patient position compared to the reference plan [2], [3], [4]. While this method is fast, easy to implement and adapts to translation and slight rotations of the patient, it does not take into account any variations in patient anatomy [5], [6]. This can only be achieved by adapting the radiotherapy plan based on the current anatomy acquired via on-board imaging such as cone-beam computed tomography (CB-CT) or magnetic resonance imaging (MRI)[7]. The acquired images must then be contoured and a treatment plan needs to be re-optimized matching this fraction’s anatomy [8].

Although online plan adaptation has the potential to yield a significant reduction in organ at risk (OAR) dose while maintaining a high dose coverage in the planning target volume (PTV), plan adaptation may lead to long treatment duration due to a time-consuming online workflow, consisting of contouring and plan re-optimization [9], [10]. To increase efficiency, simplifications to speed up the planning process have been proposed and implemented by several groups [11], [12]. By generating a reference plan prior to the first treatment fraction and adapting it based on the daily anatomy, a reduction in online planning time could be achieved. The time reduction crucially depends on the number of treatment plan changes to adapt the reference plan [13], [14]. The more the reference plan is adapted the longer takes the re-optimization process [15], [16]. In most clinical situations today, the reference plan is adapted based on the daily segmentation to adjust the delivered dose to the current position and volume of the targets and OARs but without changing the plan constraints for the optimization [17], [18], [19]. This leads to a workflow duration of about 30–60 min which has been reported to be tolerated well by patients and to reduce intrafraction motion compared to full re-optimization [19], [20], [21], [22], [23]. Even though time-efficient, this method does not allow to adapt volume-dependent plan constraints that are sensible to the anatomic changes related to volume growth or shrinkage or organ filling. Especially in abdominal and pelvic tumors where large anatomic variance is prevalent due to bladder and bowel filling, misrepresentation of the optimal dose distribution by the plan constraints might lead to suboptimal plans [18], [24]. For rectal cancer, Jagt et al. [25] showed that plan adaptation without constraint adaptation is feasible for similar anatomies, but substantial differences between daily anatomy and the reference plan make an adaptation of plan constraints necessary to fulfill all institutional dose-volume criteria.

Although not yet widely implemented in a clinical setting, automated treatment planning may be highly beneficial as it has been shown to be time efficient and created highly standardized planning results [26]. Thus, using automated treatment planning for online adaptive radiotherapy has the advantage of efficient planning and eliminates inter-planner variability [27], [28], [29].

The aim of this study was to investigate the differences in terms of plan quality during online adaptive radiotherapy when using complete re-optimization including constraint adaptation in comparison to a time-efficient re-planning based on the reference plan and the actual clinical plans. Additionally, we aimed to identify anatomic patterns that determine if complete re-optimization including plan constraint adaptations are necessary.

## 2 Materials and methods

### 2.1 Patient data

Computed tomography (CT) and MRI data of ten prostate cancer patients with localized disease and an indication for primary radiotherapy treated at a 1.5 T MR-Linac (Unity, Elekta AB, Sweden) were used for this retrospective analysis. All patient data were part of a phase II trial approved by the internal review board (NCT041722753) and patients gave written informed consent to the academic use of trial data.

The PTV57.6 consisting of prostate and seminal vesicles plus a safety margin of 6 mm / 5 mm (dorsal) was prescribed a dose of 20 x 2.88 Gy. Simultaneously, the PTV60 defined as prostate plus a safety margin of 5 mm / 0 mm (dorsal) was irradiated to a total dose of 20 x 3 Gy. In clinical practice, depending on the similarity to the reference plan anatomy, the plan was either applied after virtual couch shift or re-optimized considering the actual anatomy. The decision between the two methods was done via visual inspection by a board-certified radiation oncologist and only fractions with adapted contours based on the daily anatomy were incorporated in this study.

### 2.2 Contouring

T2 weighted turbo spin echo (TSE) sequences were used in all patients following a standard clinical template except for one patient where a higher acquisition resolution was used (Supplementary Table S1). In the clinical MR-Linac workflow OARs were not fully re-contoured due to limited time and were thus only adapted 3 cm around the PTVs based on contours propagated from the reference structure set [12]. In some fractions, this led to contours with large discrepancies from the actual underlying volumes of the daily MRI.

To acquire contours that accurately match the daily MRI, a commercial deep learning-based segmentation software (ART-Plan V1.10.0, TheraPanacea, Paris, France) was used to contour all OARs except the external patient contour and reviewed by a medical physicist. The accuracy of the software to segment the external patient contour was not high enough to be used for this study. Consequently, external patient contour and both PTVs were copied from the actual adapted treatment plan which were defined and approved by board-certified radiation oncologists. For the dose calculation based on the MRI, a population-based bulk electron density override approach was used. An organ-specific electron density was assigned to each OAR and bone based on the results of Coric et al. [30]. In total, 144 adaptive fractions were included in this study with a median of 14.5 adaptive fractions per patient (range: 7–20). In seven of these fractions manual re-contouring of the bladder was necessary as automatic segmentation failed. Each of these were fractions belonging to the same patient, where a different MRI sequence had been used (Supplementary Table S1).

### 2.3 Treatment planning

Automated treatment planning was implemented using particle swarm optimization (PSO), as described previously in detail by Künzel et al. [31], [32], [33]. Plans were generated for a 1.5 T MR-Linac (Unity, Elekta AB, Sweden) using a 9-beam step and shoot intensity modulated radiotherapy technique with a maximum of 60 segments. A total of four organ constraints, three rectum and one bladder constraints, were optimized based on daily patient anatomy using the automatic PSO algorithm (cf. Supplementary Table S2). All other constraints like maximum dose constraints or quadratic overdose penalties remained unchanged during PSO.

First, a reference plan was created for every patient based on the planning CT. To achieve optimal planning results, the PSO algorithm calculated 4800 possible plan configurations. For plan results within 120 min, the PSO ran on a high-performance computing cluster (bwforCluster NEMO). Second, for every treatment fraction where contour adaptation was performed three separate treatment plans were evaluated:

(1) Reference approach: re-planning on the daily anatomy with constraints identical to the reference plan (calculation time < 15 min).
(2) PSO approach: complete plan re-optimization based on daily MRI using automatic PSO-based treatment planning with 1600 plan configurations and the reference constraints as input for faster convergence to the optimal configuration (calculation time of < 120 min).
(3) Clinical plans: clinically delivered plans optimized based on the clinical structure set. Similar workflow to the reference approach, but with manual plan adaptation by planners if needed.

Final segmentation and dose calculations were performed using the treatment planning system (TPS) Monaco V5.51.11 (Elekta AB, Stockholm, Sweden), while no plan constraint adaptation was performed. A 3 mm dose grid size was used with a statistical uncertainty of 1 % for all plan calculations.

### 2.4 Institutional dose-volume criteria

Treatment plan quality was evaluated based on 14 dose-volume criteria specified in our institutional standard operation procedure (cf. Table 1). Five criteria scored the target volumes while the remaining nine were associated with OARs. The criteria consisted of 13 dose volume histogram (DVH) based criteria and one geometric criterion determining the maximum rectal circumferential dose. For analysis, DVH and dose files of all fractions were exported and processed in Matlab 2021b. Total adherence to the criteria was compared using a one-sided Wilcoxon signed rank test with a significance level of α = 0.05. Six criteria were plotted as cumulative histograms consisting of at least one histogram for each structure in addition to the criteria with the lowest adherence. To compare treatment time and plan complexity total number of segments and monitor units (MU) was compared for every fraction.

**Table 1:** Instituional planning objectives for prostate cancer radiotherapy (20 x 3 Gy):

Volume of interest	Dose-volume criterion
PTV60	D98% > 57 Gy
	D50% > 60 Gy
	D2% < 64.2 Gy
PTV57.6	D98% > 54.7 Gy
	D50% > 57.6 Gy
Rectum	D0.01 % < 60 Gy
	V50 Gy < 22 %
	V40 Gy < 38 %
	V30 Gy < 57 %
	V20 Gy < 85 %
	circumferential dose < 30 Gy
Bladder	V60 Gy < 2 %
	V56 Gy < 18 %
	V48 Gy < 50 %

A Spearman correlation (α = 0.05) was utilized to investigate the correlation between the different criteria and volumetric changes in targets, rectum and bladder. Due to clinical plans being calculated based on a different structure set the impact of volumetric changes was only investigated for the PSO and reference approach. The Spearman correlation coefficients were graded based on Chan et al. [34]. To compare fractions with low target coverage the 10th quartile was calculated for PTV57.6 D98%.

## 3 Results

A total of 32 out of 144 fractions (22 %) met all criteria when using the reference approach in contrast to 45 fractions (31 %) with the PSO approach and 16 fractions (11 %) in clinical plans. The highest difference between the three approaches was observed for plans which failed to fulfill at most one dose-volume criteria, which were 81 (56 %) when using the reference approach, 107 (74 %) for PSO approach and 59 fractions for clinical plans (Table 2). Only the reference approach had a fraction fail more than four criteria. Based on a one-sided Wilcoxon signed rank test a full re-optimization with the PSO approach showed a significant increase in adherence compared to the reference approach (p < 0.001) and clinically delivered plans (p < 0.001). Additionally, the reference approach showed higher adherence than the clinical plans (p < 0.001).

**Table 2:** Adherence to clinical criteria by PSO approach, Reference approach and clinical plans. Relative Adherence is indicated by values in brackets. Results with an adherence below 95% are highlighted in bold.

Structure	Criteria	Reference	PSO	Clinic
PTV60	D98%	**116 (81 %)**	137 (95 %)	**122 (85 %)**
	D50%	**126 (87 %)**	**124 (86 %)**	**37 (26 %)**
	D2%	144 (100 %)	144 (100 %)	144 (100 %)
PTV57.6	D98%	**87 (60 %)**	**97 (67 %)**	**80 (56 %)**
	D50%	144 (100 %)	144 (100 %)	144 (100 %)
Rectum	D0.01 %	**77 (53 %)**	**75 (52 %)**	**79 (55 %)**
	V50Gy	142 (99 %)	144 (100 %)	144 (100 %)
	V40Gy	144 (100 %)	144 (100 %)	144 (100 %)
	V30Gy	144 (100 %)	144 (100 %)	144 (100 %)
	V20Gy	144 (100 %)	144 (100 %)	144 (100 %)
	Circ 30 Gy	**117 (81 %)**	137 (95 %)	139 (97 %)
Bladder	V60Gy	144 (100 %)	144 (100 %)	144 (100 %)
	V56Gy	144 (100 %)	144 (100 %)	144 (100 %)
	V48Gy	144 (100 %)	144 (100 %)	144 (100 %)
Total Adherence	14 Criteria	**32 (22 %)**	**45 (31 %)**	**16 (11 %)**
	13 + Criteria	**81 (56 %)**	**107 (74 %)**	**59 (41 %)**
	12 + Criteria	**127 (88 %)**	**133 (92 %)**	**101 (70 %)**
	11 + Criteria	137 (96 %)	143 (99 %)	**136 (94 %)**
	10 + Criteria	143 (99 %)	144 (100 %)	144 (100 %)

**Fig. 1:**
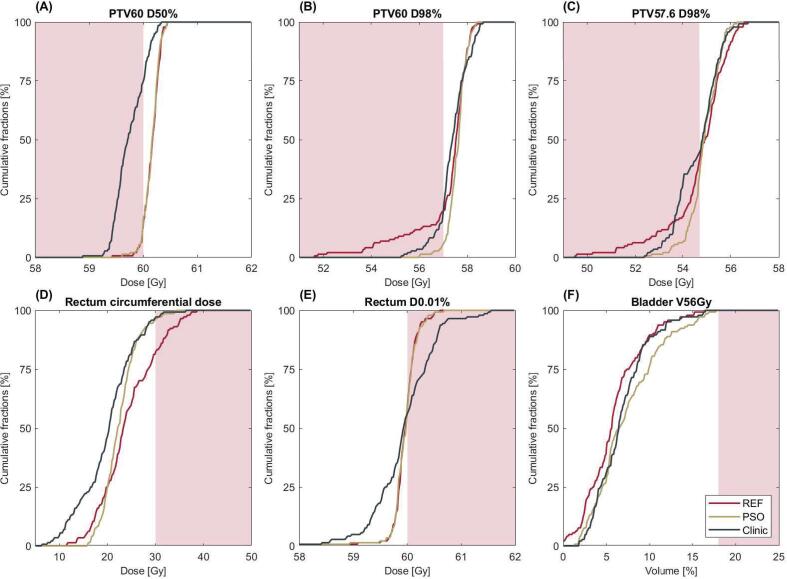
Cumulative histograms for dose-volume criteria of the PSO (beige), reference (red) approach and clinical plans (black) for: (A) PTV60 D50% (B) PTV60 D98% (C) PTV57.6 D98% (D) Rectum circumferential dose (E) Rectum D0.01 % (F) Bladder V56 Gy. Criteria failure regions are labeled in red. (For interpretation of the references to colour in this figure legend, the reader is referred to the web version of this article.). (For interpretation of the references to colour in this figure legend, the reader is referred to the web version of this article.)

In Fig. 1 the results of six exemplary dose-volume criteria are shown. For PTV60 D50% reference and PSO approach showed similar distributions with over 124 fractions adhering to the criteria compared to 37 fractions in clinical plans. The reference approach fulfilled the institutional dose-volume criteria for PTV60 D98% in 116 fractions (81 %) compared to 137 fractions (79 %) with a PSO approach and 122 clinical plans (85 %) (Fig. 1B). The histogram of PTV57.6 D98% shows a similar behaviour with dose-volume criteria being met in 87 (60 %) compared to 97 (67 %) and 80 (56 %) fractions for reference approach, PSO and clinical plans (Fig. 1C). The 10th quartile of PTV60 D98% was below 55.6 Gy in the reference compared to 57.2 Gy in the PSO approach and 56.7 Gy in clinical plans. Out of the six criteria associated with rectum complications, four could be fulfilled in all fractions but two. However, maximum and circumferential dose criteria of the rectum were not respected in multiple fractions as demonstrated in Fig. 1D/E. While for the maximum dose, there was no difference observable between the PSO and reference approaches, the clinical plans showed a more diverse distribution with fractions reaching maximum doses between 58.5 and 61.5 Gy. For the maximum circumferential rectum dose, the reference approach showed the highest doses with 117 fractions (81 %) adhering to the criteria in comparison to 137 (95 %) and 139 (97 %) for PSO approach and clinical plans. For bladder dose-volume criteria, re-optimization led to higher doses while still reaching the criteria in all fractions.

The median number of segments varied only slightly with 54, 55 and 58 segments for reference, PSO approach and clinical plans. The reference approach used a median of 529 MU in comparison to PSO with 542 MU and the clinical plans with 592 MU.

**Fig. 2:**
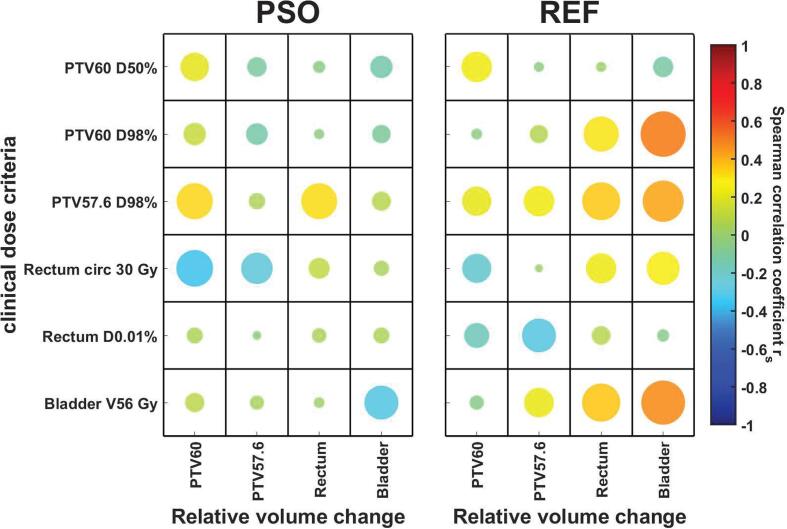
Spearman correlation between dose-volume criteria and relative volume change. The dot size correlates with the absolute value of the spearman correlation coefficient r_s_.

For PSO-based plan adaptation, fair negative correlations were observed between changes in bladder volume with respect to the reference plan and bladder criteria (Fig. 2). Moreover, a fair correlation of r_s_ = 0.33 was identified between PTV57.6 D98% and the PTV60 as well as rectum volume. The reference approach showed a fair correlation (r_s_ = 0.52) between the relative bladder volume change and PTV60 D98%. Plan constraints for PTV57.6 D98% and Bladder V56 Gy were similarly fair correlated (r_s_ = 0.43, r_s_ = 0.5) to the bladder volume. Only a poor correlation between rectal circumferential dose and relative volume change of the rectum could be found (r_s_ = 0.23).

**Fig. 3:**
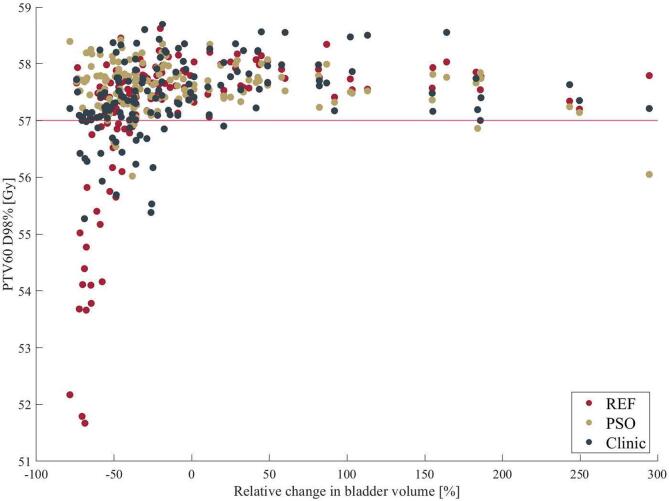
PTV60 D98% as a function of relative bladder volume change for PSO-, reference approach and clinical fractions. The criteria boundary was labeled in red at 57 Gy. (For interpretation of the references to colour in this figure legend, the reader is referred to the web version of this article.). (For interpretation of the references to colour in this figure legend, the reader is referred to the web version of this article.)

In order to investigate the sensitivity of target coverage with respect to changes in bladder volume, the correlation between PTV60 D98% and relative bladder volume change was visualized in Fig. 3. Both investigated approaches showed a strong decrease in PTV60 D98% for bladder volumes decreasing by 50 % or more with respect to the volume in the reference plan. The PSO approach maintained target coverage at a minimum of 56 Gy, whereas the minimum was lower than 52 Gy for the reference approach.

**Fig. 4:**
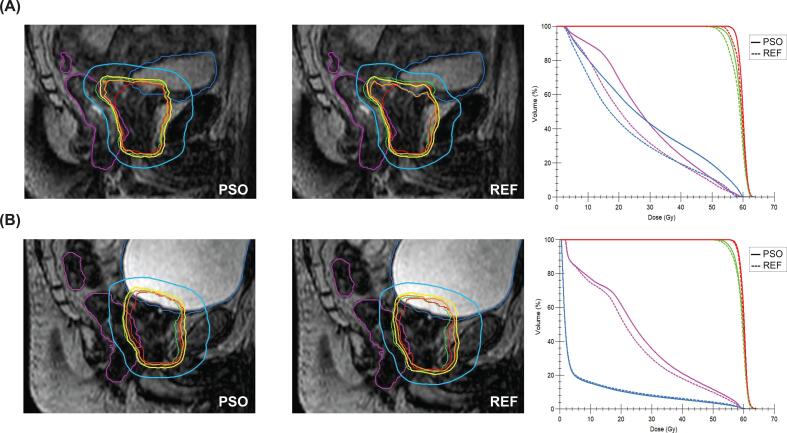
Comparison of PSO and reference approach for large bladder volume changes. DVH graphs and sagittal isocenter slices for two fractions of the same patient (P08) with (A) large decrease (-75 %) (B) increase of bladder filling (+107 %) compared to the reference. Isodose lines for 57 Gy (orange), 54.7 Gy (yellow) and 30 Gy (blue) are displayed in all sagittal slices. The contours for PTV60 (red), PTV57.6 (green), Rectum (purple) and Bladder (blue) are shown. DVH lines resulting from the reference approach are represented by a dotted line, complete PSO-based re-optimization is shown as solid line. (For interpretation of the references to colour in this figure legend, the reader is referred to the web version of this article.). (For interpretation of the references to colour in this figure legend, the reader is referred to the web version of this article.)

As an example, dose distributions of two different fractions with different bladder fillings are displayed in Fig. 4. In the fraction of Fig. 4A, where a large decrease in bladder and rectum volume was observed, adapting the constraints based on the PSO outputs led to higher target coverage and higher doses in rectum and bladder criteria compared to the reference approach, while still fulfilling the institutional dose-volume criteria. In contrast, in Fig. 4B a large bladder increase led to high doses in the bladder, as the bladder V56 Gy increased by 19 % when using the reference approach from 2 % to 2.4 %.

## 4 Discussion

In this study we investigated the impact of daily plan adaptation using full PSO-based re-optimization regarding plan quality compared to a simpler adaptive approach using fixed plan constraints defined during reference planning for prostate cancer. We were able to show that by full re-optimization including adaptation of plan constraints, the adherence to our institutional dose-volume criteria could be increased significantly compared to the reference approach and clinical plans.

While total adherence was significantly higher for the reference approach than in clinically delivered plans, when criteria were not met, it was by a greater margin as shown by the 10th quartile of PTV60 D98%. Low doses in PTV60 D98% were correlated with a volumetric change in bladder volume, suggesting that the bladder constraint conflicted with target constraints. This conflict was particularly evident in fractions with bladder volumes below 50 % of the volume in the planning CT. A similar effect was observed in the clinical plans, although less pronounced as planners on-site adapted constraints based on their clinical experience. Thus, a PSO-informed plan constraint adaptation seems to be advisable for bladder volume decreases below 50 % to aid the planner in achieving consistently high dose coverage. Besides bladder and PTV dose-volume criteria, the PSO approach and clinical plans achieved lower circumferential rectum doses than the reference approach, indicating a sensitivity to anatomic variation. A poor correlation between rectum volume change and circumferential dose seems to suggest that the circumferential isodose line not only depends on volume, but requires additional spatial information such as axial extensions of the rectum contour.

In most online adaptive workflows for MR-guided radiotherapy contours are adapted manually with optional support through rigid or deformable registration [20], [35], [36]. To shorten effective contouring time certain simplifications like manual contour correction only 3 cm around PTVs are used leading to only allow absolute volume criteria for plan evaluation [12], [20], [37], [38]. Even though these simplifications may lead to fast treatment times, contours used for plan adaptation will not represent the true underlying anatomy and may often be influenced by the pre-existing reference contours [35], [39], [40]. This might lead to inconsistent dose distributions and DVHs which may not mirror the actual dose received by the true anatomical OARs. Cropping OARs to high-dose areas to increase the robustness of the optimization was infeasible due to the usage of biological optimization functions. Deep learning based automated segmentation as used in this study can lead to a more accurate representation of the actual dose distribution in DVHs at the cost of an increase in contouring time and segmentation variance compared to propagating contours [41], [42].

Jagt et al. [25] used automated treatment planning in a similar study to benchmark daily plan adaption for rectal cancer. A marginal increase in plan quality could be achieved by adapting plan constraints via automated planning software. However, no need for daily plan adaptation including plan constraint adaption was found, as non-adapted plan constraints achieved clinically acceptable plans in 69/74 fractions. For those fractions without clinically acceptable plans, large anatomic differences between reference and fraction were identified as the cause of low plan quality. The difference between this study and our results is likely due to smaller anatomic changes in neighboring OARs in rectal cancer in combination with a more stable OAR volume because of combining the bladder and bowel structures into one large OAR structure. The results of our study confirm that an implementation of thresholds in terms of volume changes for plan adaptation is essential to increase workflow efficiency and plan quality in difficult anatomies.

The automatic treatment planning software based on PSO which was used in our study is currently not part of a clinical online workflow for MR-guided radiotherapy. This is due to computation times of up to 120 min as well as regulatory aspects. Naccarato et al. showed promising results using mCycle, a multicriterial optimization algorithm for automatic planning, reaching mean optimization times of 8 min for prostate cancer plans [26]. mCycle showed an increase in target dose coverage with concurrently reduced rectal dose. An implementation of this method in the clinical workflow might allow fast plan adaptation even for difficult anatomies without manually adapting plan constraints. To decrease calculation times in the PSO approach the number of calculated plans has to be reduced thus defeating its purpose of convergence. A feasible substitute to the PSO approach would be a dose prediction based on neural networks. Making use of consistent plan quality in the PSO approach, a robust dose prediction tool for plan constraint estimation in minutes may be possible in the future, similar to Nguyen et al. [43]. All findings were made based on the workflow of MR-guided radiotherapy, they can nevertheless be translated to CB-CT based adaptive planning due to the general workflow and mechanisms being similar.

In conclusion, fully automatic re-optimization per fraction allows for optimal target coverage and OAR sparing even in cases with large organ volume changes. A stable plan quality can be achieved using the reference plan constraints for fractions with small anatomic changes. For large anatomic changes like a reduction of bladder volume by half, plan adaptation with plan constraint adaptation is recommended to ensure high plan quality. While this has to be done manually in the current clinical setting of online adaptive radiotherapy, fast automatic treatment planning might play an important role towards real-time adaptive radiotherapy in the future.

## 5 Financial support

This study received funding by the German Research Foundation (DFG Grants No. ZI 736/2-1).

## CRediT authorship contribution statement

**Benjamin Tengler:** Software, Resources, Investigation, Data curation, Writing – original draft. **Luise A. Künzel:** Software, Resources, Data curation, Writing – review & editing. **Markus Hagmüller:** Software, Resources, Data curation, Writing – review & editing. **David Mönnich:** Resources, Data curation, Investigation, Writing – review & editing. **Simon Boeke:** Investigation, Data curation, Resources, Writing – review & editing. **Daniel Wegener:** Investigation, Data curation, Resources, Writing – review & editing. **Cihan Gani:** Investigation, Data curation, Resources, Writing – review & editing. **Daniel Zips:** Conceptualization, Funding acquisition, Writing – review & editing. **Daniela Thorwarth:** Conceptualization, Supervision, Funding acquisition, Project administration, Writing – review & editing.

## Declaration of competing interest

The authors declare the following financial interests/personal relationships which may be considered as potential competing interests: The Department of Radiation Oncology Tübingen receives within the frame of research agreements financial and technical support as well as sponsoring for travels and scientific symposia from Elekta AB (Stockholm, Sweden), TheraPanacea (Paris, France), Philips GmbH (Best, The Netherlands); Dr. Sennewald Medizintechnik GmbH (Munich, Germany), PTW (Freiburg, Germany), Siemens (Munich, Germany) and Kaiku Health (Helsinki, Finland).

## References

[1] Jaffray D.A. (2012). Image-guided radiotherapy: from current concept to future perspectives. Nat Rev Clin Oncol.

[2] Verellen D., Ridder M.D., Linthout N., Tournel K., Soete G., Storme G. (2007). Innovations in image-guided radiotherapy. Nat Rev Cancer.

[3] Ghadjar P., Fiorino C.a.f., Rosenschöld P.M., Pinkawa M., Zilli T., van Der Heide U.A. (2019). ESTRO ACROP consensus guideline on the use of image guided radiation therapy for localized prostate cancer. Radiat Oncol.

[4] Hall W.A., Paulson E., Li X.A., Erickson B., Schultz C., Tree A. (2022). Magnetic resonance linear accelerator technology and adaptive radiation therapy: An overview for clinicians. CA Cancer J Clin.

[5] Van Herk M. (2007). Semin Radiat Oncol.

[6] Peng C., Ahunbay E., Chen G., Anderson S., Lawton C., Li X.A. (2011). Characterizing interfraction variations and their dosimetric effects in prostate cancer radiotherapy. Int J Radiat Oncol Biol Phys.

[7] Choudhury A., Budgell G., MacKay R., Falk S., Faivre-Finn C., Dubec M. (2017). The future of image-guided radiotherapy. Clin Oncol (R Coll Radiol).

[8] Acharya S., Fischer-Valuck B.W., Kashani R., Parikh P., Yang D., Zhao T. (2016). Online magnetic resonance image guided adaptive radiation therapy: first clinical applications. Int J Radiat Oncol Biol Phys.

[9] Finazzi T., Palacios M.A., Spoelstra F.O., Haasbeek C.J., Bruynzeel A.M., Slotman B.J. (2019). Role of on-table plan adaptation in MR-guided ablative radiation therapy for central lung tumors. Int J Radiat Oncol Biol Phys.

[10] Henke L., Kashani R., Robinson C., Curcuru A., DeWees T., Bradley J. (2018). Phase I trial of stereotactic MR-guided online adaptive radiation therapy (SMART) for the treatment of oligometastatic or unresectable primary malignancies of the abdomen. Radiat Oncol.

[11] Glide-Hurst C.K., Lee P., Yock A.D., Olsen J.R., Cao M., Siddiqui F. (2021). Adaptive radiation therapy (ART) strategies and technical considerations: a state of the ART review from NRG oncology. Int J Radiat Oncol Biol Phys.

[12] Bohoudi O., Bruynzeel A.M., Senan S., Cuijpers J.P., Slotman B.J., Lagerwaard F.J. (2017). Fast and robust online adaptive planning in stereotactic MR-guided adaptive radiation therapy (SMART) for pancreatic cancer. Radiat Oncol.

[13] Eckl M., Sarria G.R., Springer S., Willam M., Ruder A.M., Steil V. (2021). Dosimetric benefits of daily treatment plan adaptation for prostate cancer stereotactic body radiotherapy. Radiat Oncol.

[14] Winkel D., Bol G.H., Kiekebosch I.H., Van Asselen B., Kroon P.S., Jürgenliemk-Schulz I.M. (2018). Evaluation of online plan adaptation strategies for the 1.5 T MR-linac based on “First-In-Man” treatments. Cureus.

[15] Magallon-Baro A., Milder M.T., Granton P.V., Nuyttens J.J., Hoogeman M.S. (2021). Comparison of daily online plan adaptation strategies for a cohort of pancreatic cancer patients treated with SBRT. Int J Radiat Oncol Biol Phys.

[16] Winkel D., Bol G.H., Kroon P.S., van Asselen B., Hackett S.S., Werensteijn-Honingh A.M. (2019). Adaptive radiotherapy: the Elekta Unity MR-linac concept. Clin Transl Radiat Oncol.

[17] van Timmeren J.E., Chamberlain M., Krayenbuehl J., Wilke L., Ehrbar S., Bogowicz M. (2020). Treatment plan quality during online adaptive re-planning. Radiat Oncol.

[18] Landoni V., Saracino B., Marzi S., Gallucci M., Petrongari M.G., Chianese E. (2006). A study of the effect of setup errors and organ motion on prostate cancer treatment with IMRT. Int J Radiat Oncol Biol Phys.

[19] Werensteijn-Honingh A.M., Kroon P.S., Winkel D., Aalbers E.M., van Asselen B., Bol G.H. (2019). Feasibility of stereotactic radiotherapy using a 1.5 T MR-linac: multi-fraction treatment of pelvic lymph node oligometastases. Radiat Oncol.

[20] Tetar S.U., Bruynzeel A.M., Lagerwaard F.J., Slotman B.J., Bohoudi O., Palacios M.A. (2019). Clinical implementation of magnetic resonance imaging guided adaptive radiotherapy for localized prostate cancer. Phys Imaging Radiat Oncol.

[21] Intven M., van Otterloo S.M., Mook S., Doornaert P., de Groot-van B.E., Sikkes G. (2021). Online adaptive MR-guided radiotherapy for rectal cancer; feasibility of the workflow on a 1.5 T MR-linac: clinical implementation and initial experience. Radiat Oncol.

[22] Paulson E.S., Ahunbay E., Chen X., Mickevicius N.J., Chen G.-P., Schultz C. (2020). 4D-MRI driven MR-guided online adaptive radiotherapy for abdominal stereotactic body radiation therapy on a high field MR-Linac: Implementation and initial clinical experience. Clin Transl Radiat Oncol.

[23] de Muinck Keizer D.M., de Groot-van Breugel E.N., Raaymakers B.W., Lagendijk J.J., de Boer H.C. (2021). On-line daily plan optimization combined with a virtual couch shift procedure to address intrafraction motion in prostate magnetic resonance guided radiotherapy. Phys Imaging Radiat Oncol.

[24] De Crevoisier R., Tucker S.L., Dong L., Mohan R., Cheung R., Cox J.D. (2005). Increased risk of biochemical and local failure in patients with distended rectum on the planning CT for prostate cancer radiotherapy. Int J Radiat Oncol Biol Phys.

[25] Jagt T.Z., Janssen T.M., Betgen A., Wiersema L., Verhage R., Garritsen S. (2022). Benchmarking daily adaptation using fully automated radiotherapy treatment plan optimization for rectal cancer. Phys Imaging Radiat Oncol.

[26] Naccarato S., Rigo M., Pellegrini R., Voet P., Akhiat H., Gurrera D. (2022). Automated planning for prostate stereotactic body radiation therapy on the 1.5 T MR-Linac. Adv Radiat Oncol.

[27] Wheeler P.A., Chu M., Holmes R., Smyth M., Maggs R., Spezi E. (2019). Utilisation of Pareto navigation techniques to calibrate a fully automated radiotherapy treatment planning solution. Phys Imaging Radiat Oncol.

[28] Roach D., Wortel G., Ochoa C., Jensen H.R., Damen E., Vial P. (2019). Adapting automated treatment planning configurations across international centres for prostate radiotherapy. Phys Imaging Radiat Oncol.

[29] Wortel G., Eekhout D., Lamers E., van der Bel R., Kiers K., Wiersma T. (2021). Characterization of automatic treatment planning approaches in radiotherapy. Phys Imaging Radiat Oncol.

[30] Coric I., Shreshtha K., Roque T., Paragios N., Gani C., Zips D. (2022). Dosimetric Evaluation of Dose Calculation Uncertainties for MR-Only Approaches in Prostate MR-Guided Radiotherapy. Front Phys.

[31] Künzel L.A., Nachbar M., Hagmüller M., Gani C., Boeke S., Zips D. (2021). First experience of autonomous, un-supervised treatment planning integrated in adaptive MR-guided radiotherapy and delivered to a patient with prostate cancer. Radiat Oncol.

[32] Künzel L.A., Nachbar M., Hagmüller M., Gani C., Boeke S., Wegener D. (2022). Clinical evaluation of autonomous, unsupervised planning integrated in MR-guided radiotherapy for prostate cancer. Radiat Oncol.

[33] Künzel L.A., Leibfarth S., Dohm O.S., Müller A.-C., Zips D., Thorwarth D. (2020). Automatic VMAT planning for post-operative prostate cancer cases using particle swarm optimization: A proof of concept study. Phys Med.

[34] Chan Y. (2003). Biostatistics 104: correlational analysis. Singapore Med J.

[35] Sritharan K., Dunlop A., Mohajer J., Adair-Smith G., Barnes H., Brand D. (2022). Dosimetric comparison of automatically propagated prostate contours with manually drawn contours in MRI-guided radiotherapy: A step towards a contouring free workflow. Clin Transl Radiat Oncol.

[36] Bertelsen A.S., Schytte T., Møller P.K., Mahmood F., Riis H.L., Gottlieb K.L. (2019). First clinical experiences with a high field 1.5 T MR linac. Acta Oncol.

[37] Corradini S., Alongi F., Andratschke N., Azria D., Bohoudi O., Boldrini L. (2021). ESTRO-ACROP recommendations on the clinical implementation of hybrid MR-linac systems in radiation oncology. Radiat Oncol.

[38] Willigenburg T., de Muinck Keizer D.M., Peters M., Claes A., Lagendijk J.J., de Boer H.C. (2021). Evaluation of daily online contour adaptation by radiation therapists for prostate cancer treatment on an MRI-guided linear accelerator. Clin Transl Radiat Oncol.

[39] Wu Q.J., Li T., Wu Q., Yin F.-F. (2011). Adaptive radiation therapy: technical components and clinical applications. Cancer J.

[40] Fransson S., Tilly D., Strand R. (2022). Patient specific deep learning based segmentation for magnetic resonance guided prostate radiotherapy. Phys Imaging Radiat Oncol.

[41] Kawula M., Hadi I., Nierer L., Vagni M., Cusumano D., Boldrini L. (2023). Patient-specific transfer learning for auto-segmentation in adaptive 0.35 T MRgRT of prostate cancer: a bi-centric evaluation. Med Phys.

[42] Nachbar M., lo Russo M., Gani C., Boeke S., Wegener D., Paulsen F. (2023). Automatic AI-based contouring of prostate MRI for online adaptive radiotherapy. Z Med Phys.

[43] Nguyen D, Long T, Jia X, Lu W, Gu X, Iqbal Z (2019). A feasibility study for predicting optimal radiation therapy dose distributions of prostate cancer patients from patient anatomy using deep learning. Sci Rep.

