# Supplementary material for: Full daily re-optimization improves plan quality during online adaptive radiotherapy
Source: Phys Imaging Radiat Oncol. 2024 Jan 10;29:100534. doi: 10.1016/j.phro.2024.100534 (PMC10827578; doi:10.1016/j.phro.2024.100534)
Supplement: Supplementary data 1 [file mmc1.docx]

Supplementary Material Table S1: MRI sequence details for both sequences used in this study. Default sequence was T2-2min, while T2-3D was only used for patient 6.

| Sequence | T2-2min | T2-3D |
| --- | --- | --- |
| FOV (AP x RL x FH) [mm] | 400 x 400 x 300 | 400 x 448 x 250 |
| Acquisition voxel size [mm] | 1.5 x 1.5 x 2 | 1.2 x 1.2 x 1.2 |
| Reconstructed voxel size [mm] | 0.83 x 0.83 x 1 | 0.52 x 0.52 x 0.6 |
| Flip angle [°] | 90 | 90 |
| TR/ TE [ms] | 1535/ 278 | 1300/ 82 |
| Scantime [min] | 1:57 | 6:51 |

Supplementary Material Table S2: Plan constraint template used for all treatment plans. Variable constraints through PSO are marked in orange.

| Structure | type | Reference dose | exponent | Shrink [mm] | isoconstraint |
| --- | --- | --- | --- | --- | --- |
| PTV60 | Target EUD |  |  |  | 60 |
|  | Quadratic Overdose | 60 |  |  | 0.8 |
| PTV57.6 | Target EUD |  |  | 0 | 57.6 |
|  | Quadratic Overdose | 60 |  | 0 | 0.15 |
|  | Quadratic Overdose | 57.6 |  | 3 | 0.6 |
| Rectum | Serial |  | 12 |  | variable |
|  | Serial |  | 4 | 0 | variable |
|  | Parallel | 30 | 4 | 0 | variable |
|  | Quadratic Overdose | 59 |  |  | 0.05 |
| Bladder | Serial |  | 8 |  | Variable |
|  | Quadratic Overdose | 59.8 |  |  | 0.04 |
| FemoralHead_R | Quadratic Overdose | 50 |  |  | 0.2 |
| FemoralHead_L | Quadratic Overdose | 50 |  |  | 0.2 |
| Urethra | Quadratic Overdose | 60 |  |  | 0.2 |
| PenileBulb | Parallel | 40 | 3 | 3 | 50 |
| patient | Quadratic Overdose | 56 |  | 0 | 0.05 |
|  | conformal |  |  |  | 0.8 |
